# Synthesis, ^68^Ga-Radiolabeling, and Preliminary *In Vivo* Assessment of a Depsipeptide-Derived Compound as a Potential PET/CT Infection Imaging Agent

**DOI:** 10.1155/2015/284354

**Published:** 2015-01-28

**Authors:** Botshelo B. Mokaleng, Thomas Ebenhan, Suhas Ramesh, Thavendran Govender, Hendrik G. Kruger, Raveen Parboosing, Puja P. Hazari, Anil K. Mishra, Biljana Marjanovic-Painter, Jan R. Zeevaart, Mike M. Sathekge

**Affiliations:** ^1^Department of Nuclear Medicine, University of Pretoria & Steve Biko Academic Hospital, Corner Malherbe and Steve Biko Road, Pretoria 0001, South Africa; ^2^School of Chemistry and Physics, Westville Campus, University Road, Westville, Durban 3630, South Africa; ^3^School of Health Sciences, Catalysis and Peptide Research Unit, E-Block 6th Floor, Westville Campus, University Road, Westville, Durban 3630, South Africa; ^4^Department of Virology, University of KwaZulu-Natal, National Health Laboratory Service, P.O. Box 1900, Westville, Durban 3630, South Africa; ^5^Division of PET Imaging & Radiochemistry, Molecular Imaging Research Centre, INMAS, Brig S. K. Mazumdar Marg, Timarpur, Delhi 110054, India; ^6^Radiochemistry Section Necsa, Building P1600, Pelindaba, Brits, North West Province, South Africa; ^7^Department of Science and Technology, Preclinical Drug Development Platform, North West University, 11 Hoffman Street, Potchefstroom 2520, South Africa

## Abstract

Noninvasive imaging is a powerful tool for early diagnosis and monitoring of various disease processes, such as infections. An alarming shortage of infection-selective radiopharmaceuticals exists for overcoming the diagnostic limitations with unspecific tracers such as ^67/68^Ga-citrate or ^18^F-FDG. We report here TBIA101, an antimicrobial peptide derivative that was conjugated to DOTA and radiolabeled with ^68^Ga for a subsequent *in vitro* assessment and *in vivo* infection imaging using *Escherichia coli*-bearing mice by targeting bacterial lipopolysaccharides with PET/CT. Following DOTA-conjugation, the compound was verified for its cytotoxic and bacterial binding behaviour and compound stability, followed by ^68^Gallium-radiolabeling. *µ*PET/CT using ^68^Ga-DOTA-TBIA101 was employed to detect muscular *E. coli*-infection in BALB/c mice, as warranted by the *in vitro* results. ^68^Ga-DOTA-TBIA101-PET detected *E. coli*-infected muscle tissue (SUV = 1.3–2.4) > noninfected thighs (*P* = 0.322) > forearm muscles (*P* = 0.092) > background (*P* = 0.021) in the same animal. Normalization of the infected thigh muscle to reference tissue showed a ratio of 3.0 ± 0.8 and a ratio of 2.3 ± 0.6 compared to the identical healthy tissue. The majority of the activity was cleared by renal excretion. The latter findings warrant further preclinical imaging studies of greater depth, as the DOTA-conjugation did not compromise the TBIA101's capacity as targeting vector.

## 1. Introduction

Radiopharmaceuticals are a powerful tool in managing patients with infectious diseases. However, discerning infection from sterile inflammation is still one of the most common problems in nuclear medicine. For this reason several radiopharmaceuticals have been studied to find the solution to this difficult situation. Commercially available radiopharmaceuticals, such as labeled leucocytes, Gallium-67- (^67^Ga-) citrate, Indium-111- (^111^In-) IgG, Technetium-99m- (^99m^Tc-) labeled ciprofloxacin, and Flouride-18- (^18^F-) FDG may result in false-positive diagnostics and, in some cases, a definite differential diagnosis between infection and aseptic inflammation cannot be achieved [1, 2]. Because some antimicrobial peptides (AMP) selectively target structures of the bacterial cell wall envelope, they have recently been investigated; preliminary data has suggested that these AMP have the potential to distinguish infection from aseptic inflammation [2, 3]. ^99m^Tc-UBI29-41 has been shown to have the ability to detect bacterial infection using SPECT in clinical trials [4] but an increase in the availability and accessibility of PET/CT facilities has sparked renewed interest in generator-based PET radiopharmaceuticals. Gallium-68 (^68^Ga), in particular, has received attention as an alternative positron emitter since it is not limited by the need for a nearby cyclotron and may be especially valuable in the imaging of infection/inflammation. ^68^Ga-labeled peptides have also become relevant for diagnostic imaging because of their favourable pharmacokinetics [5]. The approach using ^68^Ga-NOTA-UBI29-41 motivated for the strategy, that specific short peptides make perfect vector molecules to detect bacteria if joined by a suitable ^68^Ga-carrier molecule such as DOTA (1,4,7,10-tetraazacyclododecane-1,4,7,10-tetraacetic acid) without compromising the compounds targeting capacity.

Depsipeptides, a class of natural antimicrobial cyclic peptides, which include one or more ester (depside) bonds as part of their amide backbone, have been characterized in many natural environments and show a wide spectrum of biological activity. Owing to this unique and stabilizing motif, depsipeptides became relevant for drug discovery [6]. One depsipeptide is depsidomycin (C_38_H_65_N_9_O_9_, molecular weight of 978 g/mol). Depsidomycin is a heptadepsi-peptide isolated from the cultured broth of* Streptomyces lavendofoliae* and exhibits significant antimicrobial and immunosuppressive activity. Recently, depsidomycin analogues have been shown to be active against both normal and multidrug-resistant strains of* Mycobacterium tuberculosis* as well [7].

At present, no approaches to radiolabeling depsidomycin-derived compounds have been found with medicinal isotopes for subsequent validation for noninvasive diagnostic imaging. In this proof of concept study, we synthesized and evaluated methods to radiolabel the depsidomycin derivative DOTA-TBIA101 with ^68^Ga. We also report in this paper the preliminary findings of the antimicrobial* in vitro *behaviour of ^68^Ga-DOTA-TBIA101 and its potential for targeting infection with noninvasive imaging in an* E. coli*-bearing mice model.

## 2. Materials and Methods

### 2.1. Chemicals, Bacteria, and Material

Chemicals for peptide synthesis (GL Biochem, Shanghai, China; Sigma-Aldrich, Kempton Park, South Africa) and DOTA-tris(*t*Bu)ester (CheMatech, Dijon, France) were purchased commercially. All other solvents and reagents were procured in the highest grade quality (Merck, Modderfontein, South Africa) and used unprocessed. The bacterial strains,* Staphylococcus aureus* (ATCC 25923) (*S. aur*) and* E. coli* (ATCC 25922) were kindly provided by the Microbiology Department, University of KwaZulu-Natal. Instant thin layer chromatography silica gel paper (ITLC-SG) (Pall Life Science, Midrand, South Africa) was utilized for analysis. A ^68^Ge-^68^Ga generator was purchased for daily ^68^Ga-elution (iThemba LABS, Somerset West, South Africa). A Bruker Autoflex III MALDI-TOF-MS (matrix-assisted laser desorption ionization-time of flight mass spectrometry) was provided by the Catalysis and Peptide Research Unit, University of KwaZulu-Natal.

### 2.2. Peptide Synthesis and DOTA Conjugation

**Figure 1:**
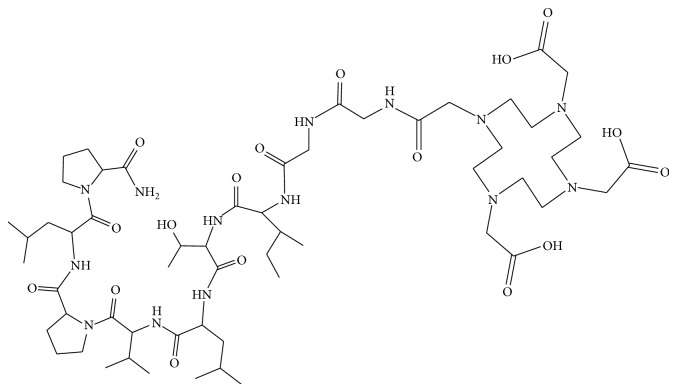
Structure of DOTA-TBIA101.

The derivative TBIA101 consists of nine amino acids (PLPVLTI-GG) with a molecular weight of 1250 g/mol. All reactions were performed under an inert atmosphere (nitrogen). The peptide synthesis (0.1 mmol) was carried out using rink amide-AM resin (0.6 mmol/g loading) on a Liberty Blue semiautomated solid-phase peptide synthesizer (CEM Corporation, Matthews, NC, USA) [7] using concentrations of amino acids,* N*,*N*-diisopropylethylamine (DIPEA), and 2-(1*H*-benzotrialzol-1-yl)-1,1,3,3-tetramethyluronium-hexa-fluorophosphate (HBTU) of 0.1, 0.4, and 0.2 mM, respectively. The on-resin conjugation of TBIA101 and DOTA-tris(*t*Bu-ester) (3 equiv) was carried out using* N*,*N*-diisopropylcarbodiimide (DIC) (3 equiv.)/Oxyma pure (3 equiv.) in* N*,*N*-dimethylformamide (DMF) (2 mL) as solvent for 2 h. The global deprotection of DOTA-TBIA101 (Figure 1) from the resin and tris(*t*Bu-ester) was accomplished within 1.5 h using a mixture of 3 mL solution of Trifluoroacetic acid/Triisopropylsilane/Water (95/2.5/2.5 v/v), cold ether was added to precipitate the peptide, centrifuged, washed twice with ether and dissolved the precipitate in double distilled water [8]. The crude DOTA-TBIA101 was used for purification.

#### 2.2.1. Purification and Analysis of DOTA-TBIA101

A reverse-phase high performance liquid chromatography (RP-HPLC), a Shimadzu 6AD instrument (Shimadzu Scientific Instruments, Kyoto, Japan), was engaged for the compound purification with an UV/VIS detector (set at 215 nm) running an ACE C18 column (150 mm × 21.2 mm × 5 *μ*) (Advanced Chromatography Technologies, Aberdeen, Scotland) at a flow rate of 15 mL/min. Buffer A consisted of 0.1% TFA/H_2_O (v/v) and buffer B consisted of 0.1% TFA/H_2_O (v/v), with a linear gradient from 0–50% B in 30 min. The peak was collected at 17 min and the molecular weight of DOTA-TBIA101 (1250 g/mol) was confirmed by a Shimadzu LCMS 2020 mass spectrometric analysis (Shimadzu Scientific Instruments, Kyoto, Japan) in the positive mode with a X-bridge C18 column (150 mm × 4.6 mm × 5 *μ*) (Waters Corporation, Eschborn, Germany).

### 2.3. MALDI-TOF-MS Analysis

MALDI-TOF-MS analysis was performed on an Autoflex III MALDI-TOF mass spectrometer (Bruker, Coventry, United Kingdom) with a 337 nm nitrogen laser operating in a linear mode at an accelerated voltage of 20 kV. The *α*-cyano-4-hydroxycinnamic acid matrix was applied: the matrix solution was prepared by making a saturated solution of the matrix powder into TA30 solution (30% ACN and 70% water containing 0.1% TFA). A MALDI plate-sandwich method was used by spotting the compounds between spots of matrix solution. Analyses were done in triplicates; spectra were collected manually and the mass peak intensities were recorded.

### 2.4. ^nat^Gallium-Complexation

The nonradioactive “cold” labeling was performed according to a described method [9]. Briefly, 100 *μ*g of gallium trichloride (pH 3.5-4) was mixed with 25 *μ*g DOTA-TBIA101 stock solution and vortexed for 30 sec. The reaction mixture was incubated for 10 min at 100°C. The crude ^nat^Ga-DOTA-TBIA101 was purified using a C_18_ Sep-pak cartridge. The cartridge was preconditioned with 2 mL of ethanol and 1 mL of water, sequentially. After passing the crude sample through the C_18_ Sep-pak, 4 mL of water was used to rinse out uncomplexed gallium. A mixture of 1% trifluoroacetic acid (TFA) in acetonitrile (ACN) (5 mL) was used to desorb ^nat^Ga-DOTA-TBIA101; its molecular weight (1319 g/mol) was confirmed using MALDI-TOF-MS.

### 2.5. Bacterial Association Assay

*S. aur* and* E. coli* strains were cultured at 37°C overnight and tested with the peptide (TBIA101), the peptide-conjugate (DOTA-TBIA101), and the gallium(III)-complexed derivative (^nat^Ga-DOTA-TBIA101). The bacterial concentration was kept at 1.5 × 10^8^ colony forming units (CFU). Five different concentrations (20, 40, 60, 80, and 100 *μ*M) of the above-mentioned compounds were used for all experiments, following the described method [10]. The final reaction volume was 600 *μ*L. Bacteria and compound mixtures were incubated at 37°C in an orbital shaker at 100 rpm for 3 h. After incubation, the cells were centrifuged at 3000 ×g for 20 min and the supernatant was removed. The bacterial pellet was washed twice with 200 *μ*L of phosphate buffered saline (pH 7.4) and 200 *μ*L of 80% ACN containing 0.1% TFA was added to dissolve the pellet followed by vigorous mixing followed by centrifugation at 3000 ×g for 20 min. The supernatant (*sample 1*) was collected, representing the membrane-associated compound fraction. The residual pellet was treated with 200 *μ*L of 80% ACN (*sample 2*), representing the cell-internalized compound fraction. Bacterial persistence in samples 1 and 2 was qualified with MALDI-TOF-MS.

### 2.6. Cytotoxicity Test

The reduction of 2,3-bis-(2-methoxy-4-nitro-5-sulfophenyl)-2-H-tetrazolium-5-carboxanilide (XTT) was used to determine the cellular cytotoxicity of TBIA101, DOTA-TBIA101, and ^nat^Ga-DOTA-TBIA101, as described by Scudiero et al. [11]. MT-4 cells were obtained through the NIH AIDS Reagent Program, Division of AIDS, NIAID, NIH: MT-4 from Dr. Douglas Richman and were cultured and all incubations were done in RPMI medium containing 10% fetal calf serum at 37°C and 5% CO_2_-atmosphere. A 6 × 10^5^ cells/mL concentration was used to seed cells into 96-well culture plates (total cell number of 2 × 10^4^ per well) followed by exposure to eight 5-fold dilutions of the stock compound solutions (1 mg/mL). The plates were incubated for 5 days before XTT was added to react for 4 h. Formazan production was quantified, measuring absorbance at 450 nm (reference wavelength 620 nm), and related to the absorbance of untreated control cells (RPMI background). Cytotoxicity was determined by extrapolating the inhibitory concentration at 50% (IC_50_).

### 2.7. ^68^Ge-^68^Ga Generator Elution

Gallium-68 was routinely obtained from a SnO_2_-based ^68^Ge-^68^Ga generator but for animal studies a TiO_2_-based ^68^Ge-^68^Ga generator (Eckert & Ziegler Isotope Products, Valencia, CA, USA) was employed. ^68^Ga-radioactivity was eluted manually using eluate volume fractionation methods as described [12, 13] and measured in a dose calibrator (CRC15, Capintec Inc, Pittsburgh, PA, USA). All ^68^Ga-activity data is expressed as decay corrected (half-life of ^68^Ga is 68 min, 88% decay by emitting positron of 1.92 MeV and 11% by electron capture).

### 2.8. ^68^Ga-DOTA-TBIA101 Radiolabeling: Optimization of Radiolabeling Conditions

Radiolabeling attempts were based on a labeling procedure described for DOTA-TATE [13]. In order to achieve efficient, high-yield ^68^Ga-labelling, the following conditions were investigated regarding an optimal ^68^Ga-DOTA-TBIA101 yield: (a) temperature influence (room temperature, 60°C and 100°C) with different incubation duration of 5–45 min, (b) influence of decreasing eluate acidity (i.e., pH values up to pH 7), and (c) influence of decreasing DOTA-TBIA101 molarity of 40–0.00128 *μ*M. The percentage labeling efficiency (%LE) and radiochemical purity (RCP) of the crude samples was determined utilizing ITLC-SG, and HPLC.

#### 2.8.1. Quality Control

The 10 × 1 cm ITLC stationary phase was spiked with a radioactive sample and exposed to the following mobile phases: (a) 0.1 M sodium citrate pH 4–4.5 (R*f* (^68^Ga) = 0.8–1.0, R*f* (^68^Ga-peptide) = 0.0–0.3) and (b) 1 M ammonium acetate/methanol (1 : 1 v/v) (R*f* (colloidal ^68^Ga) = 0.0–0.2, R*f* (^68^Ga-peptide) = 0.8–1.0). Peaks were identified and compared by region-of-interest (ROI) analysis. HPLC analysis was conducted as described [14]. Radiochemical purity was determined using a Symmetry C 18 column (4.6 mm × 250 mm × 5 *μ*) (Waters Corporation, Milford, MA, USA) coupled to 6100 quadruple MS detector (Agilent Technologies, Santa Clara, CA, USA) diode array detector and Gina Star radioactive detector (Raytest Isotopenmessgeräte, Straubenhardt, Germany) using 0.1% TFA in water (solvent A) and 0.1% TFA in ACN (solvent B) as a mobile phase. Gradient elution was carried out at 40°C with a 1.0 mL/min flow rate using 0–2 min (5% B), 2–32 min (65% B), and 32–35 min (5% B).

#### 2.8.2. Routine Labeling Method for ^68^Ga-DOTA-TBIA101

For routine radiolabeling, 1.5 mL eluted activity was directly added to the reaction mixture (pH 3–3.5 buffered using 2.5 M sodium acetate, DOTA-TBIA101: 40 *μ*M) and incubated for 15 min at >95°C. The reaction mixture was purified using a Sep-pak C_18_ cartridge to allow for uncomplexed radioactive gallium and traces of ^68^Ge to rinse off with saline solution. Desorption of the labeled product was performed with a 50% ethanolic saline solution and was aseptically filtered (low protein binding filter) before tracer administration.

#### 2.8.3. Radiochemical Integrity and Blood Stability

The radiochemical integrity determination of the final product was performed at 0, 30, 60, 120, and 180 min. The ^68^Ga percentage of unbound and bound ^68^Ga-DOTA-TBIA101 was analyzed by ITLC as described earlier. ^68^Ga-DOTA-TBIA101 stability was determined on whole blood, plasma, and serum. The blood sample (50 mL) was collected with ACD anticoagulant, 10 mL was kept for blood assessment and the remainder was allowed to separate for 20–30 min followed by plasma collection after centrifugation at 1500 rpm for 10 min. Serum was collected similar to plasma from a blood sample collected without anticoagulant. All samples were used immediately and tests were conducted in triplicate; 1 mL of ^68^Ga-DOTA-TBIA101 (38–50 MBq) was incubated with 1 mL of whole blood, plasma, or serum and analyzed by ITLC.

### 2.9. Small Animal *μ*PET/CT Imaging

Animal studies were conducted according to the guidelines of the Institute of Nuclear Medicine & Allied Sciences (INMAS) Animal Ethics Committee (CPCSEA Registration no.8/GO/a/99). Immune competent BALB/c mice (male, 26–30 g, 6–8 weeks old) were used for the study and allowed water and food* ad libitum* for the duration of the study. A 0.2 mL aliquot of viable* E. coli* (5 × 10^8^ CFU/mL) was inoculated into the right hind thigh muscle and allowed to form the infection site for 4-5 days.

#### 2.9.1. Image Acquisition, Reconstruction, and Quantification

Mice were injected with ^68^Ga-DOTA-TBIA101 intravenously into the tail vein in a single bolus of 0.1-0.2 mL tracer solution. All animals were anesthetized by injection of a mixture of 10 mg/kg xylazine (Xylavet, Kempton Park, South Africa) and 80 mg/kg ketamine (Anaket V, Centaur Laboratories, Isando, South Africa) before they were placed on the scanner bed in the prone position (head first). CT scans and PET images were acquired at 25 min after injection (whole body images or single field-of-view) of ^68^Ga-DOTA-TBIA101 in list mode. All acquired images were scatter- and transmission-corrected (CT-based) and reconstructed by the ordered-subsets expectation maximization (OSEM) algorithm, yielding 3D iterative PET/CT overlay images in axial, sagittal, and coronal orientation. The tracer distribution was determined with three-dimensional volume of interest (VOI) areas surrounding (a) whole body; (b) background, and (c) all tracer target organs such as heart, liver, kidneys, urinary bladder, lung, and noninfected and infected muscles. ^68^Ga-DOTA-TBIA101 organ distribution, represented by percentage of injected dose (%ID) calculation, and ^68^Ga-DOTA-TBIA101 concentration, represented by the calculation of standardized uptake values (SUV), were performed from the same VOI of areas.

### 2.10. Statistical Analysis

Unless stated otherwise, data was expressed as mean and standard error of mean (SEM) using Microsoft Excel Software. The significance of a statistical difference between two mean values was calculated by a* Student's t-test*. The level of significance was set at *P* ≤ 0.05.

## 3. Results

### 3.1. Synthesis of the DOTA-TBIA101

The linear DOTA-TBIA101 was synthesized by a solid phase method on a rink amide resin. DOTA was protected with a tris(*t*Bu)-moiety at three of its four carboxyl groups. The fourth unprotected carboxyl group was used to form a stable amide bond between the DOTA and the peptide's N-terminus. The DOTA-TBIA101 (Figure 1) was purified by reversed-phase-HPLC and resulted in >99% purity and the correct molecular weight of 1250 g/mol was confirmed by LC-MS and MALDI-TOF-MS for DOTA-TBIA101.

### 3.2. ^nat^Ga-DOTA-TBIA101 Complexes

The labeling experiments were initially done using gallium trichloride to determine radiolabeling conditions that would avert unnecessary exposure to radiation. The molecular weight (1319 g/mol) of ^nat^Ga-DOTA-peptide was established with MALDI-TOF-MS, confirming the complexation of gallium to DOTA-TBIA101. Post-C_18_ Sep-pak cartridge ^nat^Ga-DOTA-TBIA101 showed >99% purity.

### 3.3. Bacterial Association Assay

The qualitative justification of the bacterial binding and internalization of all relevant compounds resulted in affinity binding constants (K) for TBIA101, DOTA-TBIA101, and ^nat^Ga-DOTA-TBIA101 which were 0.022 ± 0.006 nM, 0.028 ± 0.006 nM, and 2.58 ± 1.04 nM for* E. coli* and 0.023 ± 0.007 nM, 0.029 ± 0.006 nM, and 2.97 ± 0.86 nM for* S. aur*. TBIA101 showed binding and internalization for both bacterial strains. DOTA-TBIA101 showed binding and no detectable internalization for both bacterial cells. A significantly higher* K* value for* S. aur* or* E. coli* was found for ^nat^Ga-DOTA-TBIA101 compared to both TBIA101 and DOTA-TBIA101.

### 3.4. Cytotoxicity Test

The normalized IC_50_ values of TBIA101, DOTA-TBIA101, and ^nat^Ga-DOTA-TBIA101 were calculated as 63.2, 42.1, and 33.7 *μ*M, respectively. These values indicated no obvious toxic effect at the concentrations tested.

### 3.5. ^68^Ge-^68^Ga Generator Elution

The SnO_2_-based generator provided 94 ± 3% and 85 ± 0.4% of the total calculated activity at day 10 and day 250, respectively. For this study, the total activities eluted from the TiO_2_-based ^68^Ge-^68^Ga generator were 125 ± 57 MBq (*n* = 3) and 1437 ± 489 MBq (*n* = 23) for SnO_2_-based ^68^Ge-^68^Ga generator (Table 1). Ninety percent to ninety-five percent of the eluted activity was obtained with fractionated elution for subsequent labeling.

### 3.6. Assessment of Radiolabeling Conditions and Routine Labeling

**Figure 2:**
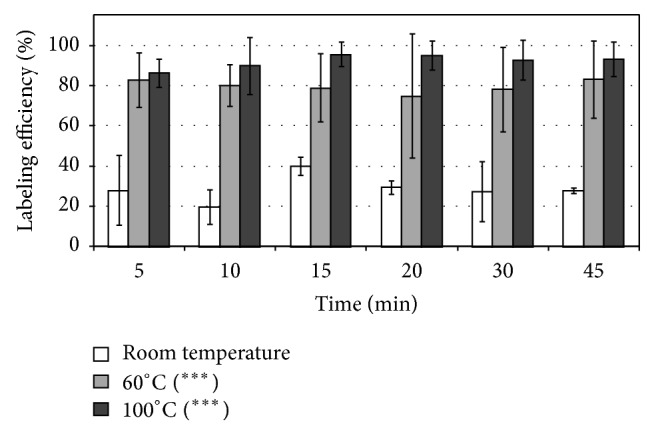
(a) Percentage labeling efficiency of ^68^Ga-DOTA-TBIA101 at different incubation temperatures, comparing room temperature (open bars), 60°C (light grey bars), and 100°C (dark grey bars) using incubation durations of 5–45 minutes. Mean and standard error of mean are presented from *n* = 6 experiments. Student's *t*-test returned a *P* value < 0.001 (^***^) when compared to values concerning incubation at room temperature for all time durations.

**Figure 3:**
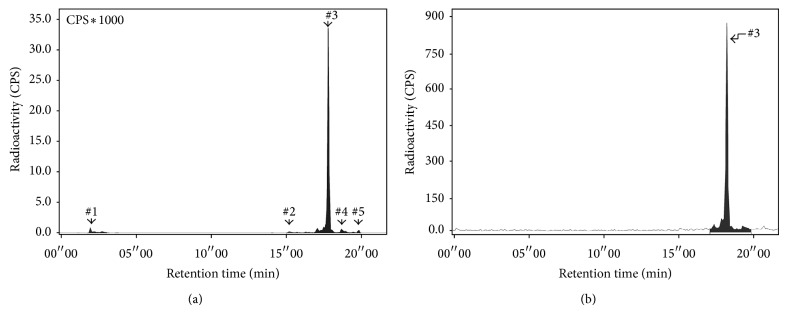
Radioanalytical HPLC chromatogram of ^68^Ga-DOTA-TBIA101 (a) before and (b) after successful C_18_ Sep-pak cartridge purification. Reg. number 3 confirmed the elution of ^68^Ga-DOTA-TBIA101 including traces of unbound ^68^Ga (Reg. number 1) and by-products (Reg. number 2, number 4, and number 5) which were successfully removed (b).

Labeling efficiency of the crude ^68^Ga-DOTA-TBIA101 labeled at room temperature, 60°C and 100°C at different time points, is presented in Figure 2. High radiolabeling was obtained after 5 min at 100°C already (range: 86–96%). A significantly lower %LE was calculated for samples incubated at 60°C compared to 100°C (*P* = 0.004, range 75–83%). The radiolabeling at room temperature (negative control) compared to 100°C and 60°C (both *P* ≤ 0.001) amounted to %LE of 28 ± 14, 19 ± 7, 40 ± 4, 30 ± 3, 27 ± 12, and 28 ± 1% for 5, 10, 15, 20, 30, and 45 min, respectively. A pH-optimum was found at 3 to 3.5, yielding a ^68^Ga-complexation of 98 ± 3% to DOTA-TBIA101. Significantly lower %LE was determined for all other pH-values [1.5 ± 0.5% (pH 1-2), 24 ± 20% (pH 4), 9 ± 3% (pH 5), 2.1 ± 1.9% (pH 6), and 1.4 ± 1.4% (pH 7)]. A DOTA-TBIA101 concentration of 40 *μ*M led to optimal ^68^Ga-complexation (92 ± 0.7%); 5- and 20-fold lower-peptide concentration yielded 44 ± 20% and 22 ± 3%; nanomolar (nM) concentration led to 15–18% %LE. Quantitative HPLC analysis of crude and pure ^68^Ga-DOTA-TBIA101 samples (40 *μ*M) showed crude radiochemical purities of bound activity to be ≥92.1% and 100% radiochemical purity (Figures 3(a) and 3(b)). In comparison the 20 *μ*M crude ^68^Ga-DOTA-TBIA101 samples showed ≤14.4%LE and ≥98.0% radiochemical purity. The labeling method caused moderate activity losses to instruments, surface material, and colloid forming (18 ± 8%).

#### 3.6.1. Routine Radiolabeling

Twenty-three radiosyntheses were routinely performed as described in a 1.34 mL reaction volume (Table 1) within 34–41 min. The desorption of ^68^Ga-DOTA-TBIA101 from the C_18_ Sep-pak cartridge using ethanol/saline mixture (1 : 1) recovered 77 ± 21% (*n* = 6). The average %LE was 64 ± 19 (*n* = 23) with good reproducibility [%LE < 25 (*n* = 1), %LE ≤ 25 ≤ 60 (*n* = 6), and %LE > 70 (*n* = 12)]. The final ^68^Ga-DOTA-TBIA101 formulation in sterile saline solution showed a pH of 5.5–6 and contained ≤ 4% ethanol. In comparison, experiments using the TiO_2_-based generator yielded a %LE of 61 and 94 ^68^Ga-DOTA-TBIA101 in 30 min.

#### 3.6.2. Quality Control

The %RCP using mobile phase 1 was detected at 99.0 ± 0.9% (*n* = 23). The R*f* values for unbound ^68^Ga and ^68^Ga-DOTA-TBIA101 were calculated 0.85–1.0 and 0.05-0.15, respectively. Using mobile phase 2, the %RCP was 99.9 ± 0.5% (*n* = 3), with R*f* values of 0.05–0.10 for colloidal ^68^Ga and 0.9-1.0 for ^68^Ga-DOTA-TBIA101. The yielded product activity was 476–856 MBq (7.7–19.5 GBq/*μ*mol).

### 3.7. Compound Integrity and Blood Stability

The RCP of ^68^Ga-DOTA-TBIA101 was found to be 95–100% over 180 min after radiolabeling (*n* = 3) and no significant unbound ^68^Ga was observed.* In vitro* stability tests showed that ^68^Ga-DOTA-TBIA101 was intact in whole blood, plasma, and serum (*n* = 3). The stability was determined by ITLC. The RCP was ≥97.2% for all time points up to 180 min at which no unbound ^68^Ga was present.

### 3.8. Small Animal PET/CT Study

^68^Ga-DOTA-TBIA101 (19 ± 11 MBq) was injected in single bolus with short-term adverse reaction observed upon injection. Image acquisition at 25 min post-injection was analysed to demonstrate tracer biodistribution (%ID) and activity concentration (mean SUV) for three infected animals and compared to one healthy animal (Table 2). In this study, no significant differences in the tracer distribution (%ID per organ or tissue) were observed: healthy animal (range: 0.45–36.5%) and infected animal (range: 0.45–36.4%) when comparing heart, lung, liver, kidneys, brain, intestines, and urinary tract including bladder as well as healthy forearm and thigh muscles. Approximately 48% of the injected radioactivity was considered excreted (represented in kidneys, urinary tract, and bladder); heart and liver showed enhanced uptake (due to perfusion) and very low activity was distributed in lung, brain, intestine, forearm muscle, and hind muscle. The SUV values reflected a similar pattern regarding the organ activity concentration compared to %ID calculation, but it was noted that most SUV values of the healthy animals were less than of the infected animals (particularly relevant for heart and liver). A higher tracer concentration (SUV = 1.3–2.4) was calculated for 3/3* E. coli*-infected muscles compared to the contralateral thigh (SUV = 1.2–2.2; *P* = 0.322; 1.21-fold), to the forearm muscles (SUV = 0.7–1.5; *P* = 0.092; 1.62-fold), or to the background (SUV = 0.66–0.78; *P* = 0.021; 2.3-fold) of the* same* animal. Normalization of the infected thigh muscle to reference tissue (forearm muscle in a healthy animal) showed a ratio of 3.0 ± 0.8 and a ratio of 2.3 ± 0.6 compared to the identical tissue (right hind thigh in the healthy animal).

**Figure 4:**
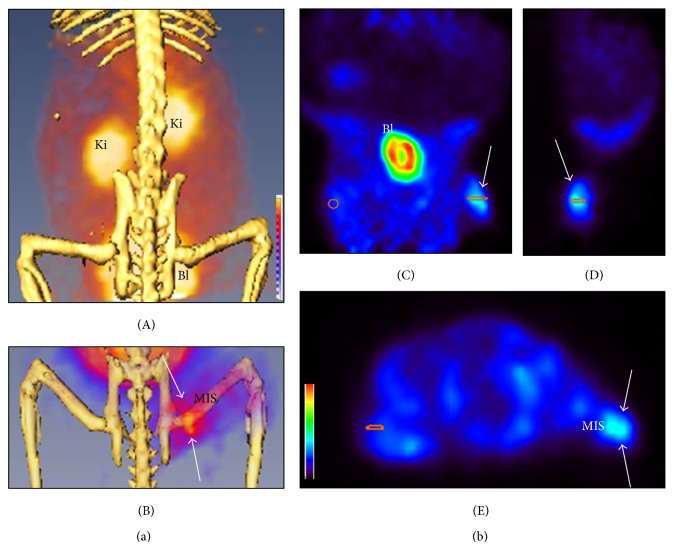
Images of BALB/c mice acquired using a Triumph *μ*PET/CT preclinical imaging system (GE Healthcare, Buckinghamshire, United Kingdom). (a) Representative *μ*PET/CT images acquired at 25 min after ^68^Ga-DOTA-TBIA101 administration, (A) maximum intensity projection (MIP) image of a healthy animal demonstrating renal excretion, (B) pelvis projection including the muscular infection site (MIS) located in the right hind muscle tissue. The arrows indicate ^68^Ga-DOTA-TBIA101 activity uptake at the site of infection. No activity uptake was noted in the contralateral muscle tissue. (b) Three-dimensional *μ*PET images show activity uptake at the infection site (indicated by the white arrows) in (C) coronal, (D) sagittal, and (E) axial orientation. Ki is Kidney and Bl is Bladder.

The reconstructed PET/CT images showed positive tracer uptake with a moderate signal-to-noise ratio and visualization of the infected target area (Figure 4(a)-B), that is, right hind muscle tissue. In 3/3 animals the infection site was clearly localized by ^68^Ga-DOTA-TBIA101, as represented in the three-dimensional slides (Figure 4(b)). No notable uptake was observed in the contralateral muscle tissue. Bacterial persistence in murine muscular tissue was not assessed with bacterial culturing postmortem. Unspecific uptake was detected in heart (blood pool) and liver and the excreted ^68^Ga-DOTA-TBIA101-related radioactivity was represented by the avid kidney and bladder signal (Figure 4(a)-A).

## 4. Discussion

Noninvasive whole body imaging technologies like PET/CT can assist in identifying infections of unknown origin or those following surgery or transplantation. However, current radiotracers that image infectious foci (e.g., radiolabeled blood elements) are host dependent, require complex procedures, or lack specificity. Although ^18^F-FDG is commonly used as imaging agent in PET and ^67^Ga-citrate in SPECT for infection/inflammation imaging, their specificity is even lower when compared with radiolabeled leukocytes. TBIA101 (PLPVLTI-GG) is a derivative of depsidomycin (PLPVLTI), an uncharged cyclic depsiheptapeptide that was extended by two glycine molecules, causing enhanced antibacterial properties. When the peptide structure is given, it is possible to design new analogues containing one or two additional glycine or alanine molecules, in order to improve efficiency against bacteria without increasing harmful side effects [15]. Three possible folding patterns can be expected: nonhydrogen, *β*-turn, and *α*-helical turn for depsipeptides. Alanine has a higher *α*-helical-forming tendency than glycine. The major conformational feature is *β*-turn, involving glycine and proline [16].

Although the mode of action for depsipeptides is not entirely understood, it is postulated that there is a possible interaction with lipopolysaccharide (LPS) structures of the bacterial cell envelope. Studies involving the related *β*-sheet peptides report effective disruption of the lipid organization and may induce lipid flip-flop or undergoing membrane translocation without causing significant calcein release from the membrane system; however no long-living pores are being formed [17]. To date, no attempts have been made to conjugate TBIA101 with DOTA to allow for complexation of the PET-radioisotope ^68^Ga. This will consequently facilitate studies of the* in vivo* distribution including targeting infectious tissue in preclinical animal models. Thus, we aimed to synthesise and radiolabel DOTA-TBIA101 with generator-eluted ^68^Ga to detect* E. coli*-based infection by *μ*PET/CT imaging of a BALB/c mice model.

As a prerequisite, the TBIA101 synthesis was successfully achieved and conjugated using DOTA-tris(*t*Bu) ester in an N-terminal peptide position based on a resin method [18]. This conjugation is limited to the N-terminal position and exhibits moderately long deprotection times for the complete cleavage of the tris(*t*Bu) ester [7]. Furthermore, DOTA-TBIA101 was nonradioactively labeled with gallium (III) trichloride to form ^nat^Ga-DOTA-TBIA101 for subsequent evaluation of its bacterial binding properties and to perform* in vitro* cytotoxicity studies whilst preventing unnecessary radiation exposure. Owing to their involvement in the innate human immune response, antimicrobial peptides are not considered cytotoxic [19], although amending the structure or conjugation and complexation with radiogallium may cause unexpected cytotoxic behavior. The IC_50_-values reported in this paper were significantly lower than DOTAVAP-P1, which is also considered for preclinical studies of inflammation and infection [20]. For the *μ*PET study, the mice were injected with approximately 500-fold less ^68^Ga-DOTA-TBIA101 (8 nM), which was well tolerable for PET/CT imaging. The administered dose of ^68^Ga-DOTA-TBIA101 is considered nontoxic for mammalian cells but also not bactericidal. Interestingly, based on MALDI-TOF-MS analysis, DOTA-TBIA101 showed a differentiated bacterial binding and a lack of internalization in both tested* S. aur* and* E. coli* strains, compared to TBIA101, despite their near-equal* K*-values. We have reason to believe that the DOTA-conjugation does not compromise the initial interaction to LPS but may affect the peptide property of initiating a membrane flip-flop mechanism, which might be due to lack of membrane occupancy with the compound, as most often a distinct threshold must be reached to alter membrane potential. It was reported that the entry into the cell by the peptides requires a minimum number, or threshold concentration, of antimicrobial peptides to accumulate on the surface of the lipid membrane. This event can be affected by factors other than concentration—such as the ability of the peptides to multimerize and also the features of the phospholipid membrane itself (e.g., its lipid composition, head group size, and fluidity) [21]. The transmembrane potential of the bilayer may also influence the way in which the peptide enters the membrane, since a highly negative transmembrane potential will facilitate membrane pore formation [22]. The ^nat^Ga-DOTA-TBIA101 also exhibited a 200-fold lower binding affinity than DOTA-TBIA101, which was unexpected but might indicate a slower pharmacodynamic once gallium is complexed. A pragmatic explanation might be the use of solvents within the preparation. All three compounds showed interaction with both bacterial cells tested and the unexpected internalization of ^nat^Ga-DOTA-TBIA101 might be due to tight integration into the cell wall. We cannot fully explain the reason for the internalization of cold labeled ^nat^Ga-DOTA-TBIA101 for both bacterial cells as compared to unlabeled TBIA101 and DOTA-TBIA101. MALDI-TOF MS has offered a highly sensitive method for mass recovery in cellular samples but more in-depth experiments with an array of known peptides would support further justification of ^nat^Ga-DOTA-TBIA(101).

As one main achievement we report the successful radiolabeling with a “high-yield”/“high-purity” approach for ^68^Ga-DOTA-TBIA101 using state-of-the-art ^68^Ge-^68^Ga-generator technology, which makes radiopharmaceutical production easy, cost efficient, and available to hospitals without access to a cyclotron infrastructure [23]. A small volume containing the majority of the eluted ^68^Ga can be used for research; however, the breakthrough of Germanium-68 (^68^Ge) and other metals or chemical impurities may hinder the complexation if the generator matrix is eluted over a prolonged duration. Consequently, we observed this direct radiolabeling method routinely and confirmed good robustness as well as high specific activities and found no relation between the %LE of ^68^Ga-DOTA-TBIA101 and the life span of the generator, which was also complemented by the low contents of competing ions, as reported previously [13, 24]. However, daily elution is required to keep the concentration of the metal ions and ^68^Ge breakthrough as low as possible and the use of a C_18_ Sep-pak cartridge is mandatory for this method before the final product is dispensed. Prepurification methods of the crude ^68^Ga eluate are available that exhibit minimal losses of activity but employ an additional cartridge purification step [25]. Our findings, as summarised in Table 1, are comparable with the previous findings reported in study that employed a SnO_2_-based generator [23, 24] and also supported the efficient separation of ^68^Ga-DOTA-TBIA101 from uncomplexed or colloidal ^68^Ga by altering polarity on the C_18_ Sep-pak cartridge unit. It has been indicated that the amount of ^68^Ge is reduced by at least 100- to 1000-fold in the final product after the C_18_ Sep-Pak cartridge has been used, but the ^68^Ge content in the final ^68^Ga preparation cannot be measured prior to tracer administration. We were able to desorb between 56% and 99% radiochemical-pure ^68^Ga-DOTA-TBIA101 as qualified and quantified by using a two-strip ITLC system or HPLC with reasonable activity losses due to the labeling protocol. ^68^Ga-DOTA-TBIA101 was found stable in human blood, serum, and plasma against transchelation over 180 min (≥97.2%) as compared to 39% plasma binding of ^68^Ga-DOTAVAP-P1 [20]. The stability of ^68^Ga-DOTA-TBIA101 was determined by ITLC as successfully carried out by other workgroup studies that examined various tracer blood stabilities [26–29] but it might lack accuracy in determining potential metabolites. Conversely, HPLC was used by Ujula et al. to assess DOTAVAP-P1 stability in human- and rat-plasma; the amount of intact product after 4 h of incubation was 88% and 87%, respectively.

The findings from a small-scale proof-of-concept experiment that set out to prove the capability of ^68^Ga-DOTA-TBIA101-PET to localize* E. coli*-infected muscle tissue after 4-5-day incubation led to contradictory results. Despite the fact that ^68^Ga-DOTA-TBIA101-PET was able to localize the infectious tissue, the authors cannot conclude that the tracer represented infection by directly targeting the bacteria. Additional results from a parallel project using the same animals returned positive visualization of the infection site with ^18^F-FDG-PET; however, ^68^Ga-NOTA-UBI29-41 was not localized congruently [30] at a late stage of infection (5-7-d). These results may raise a question on the infection-specific uptake observed with ^68^Ga-DOTA-TBIA101 or ^18^F-FDG. Except from a significant signal-to-noise ratio (*P* = 0.021), ^68^Ga-DOTA-TBIA101-PET amounted in a low T/NT SUV ratio (1.2 ± 0.1) if one compares infected muscle to the uninfected contralateral muscle tissue of the same animal. The latter T/NT ratio contradicts the study performed by Akhtar et al. using dual-time point SPECT with ^99m^Tc-UBI29-41 in* E. coli*-infected rabbits (T/NT =1.5 ± 0.4 at 30 min and 1.7 ± 0.4 at 60 min p.i.) [31]. Dual time point imaging protocol including a rescan at ≥60 min p.i. would have indicated whether the early-onset tracer uptake can be verified as target-specific accumulation. Initial uptake could be also caused by inflammatory processes or enhanced perfusion. Some compromising limitations have revealed themselves after this preliminary infection-imaging study was conducted. Experiments of greater depth and including positive controls are required to clarify outstanding matters regarding the* in vivo* performance of ^68^Ga-DOTA-TBIA101. Imaging evaluation of sterile inflammatory processes would help to cast doubt on tracer specificity particularly. The use of a well-understood infection model, including the type of bacteria and incubation duration up to PET/CT imaging, could be helpful in accommodating a certain imaging setup. Supporting imaging results with postmortem histopathology and bacterial recovery from infectious tissue may aid interpretation of noninvasive findings in future studies. For example, ^68^Ga-DOTAVAP-V1 studies were carried out two days after bacilli injection with 2-fold more injected bacteria [20]. The infected muscle to background ratio of ^68^Ga-DOTA-TBIA101 (2.3 ± 0.3) was in the same range as reported by Ujula et al. (2.3 ± 0.7) and lower compared to ^18^F-FDG (3.1 ± 0.6) for infection imaging but important control measures were carried out by the authors. In this paper %ID and SUV calculation reported on showed most of the activities recovered in the kidneys and urinary tract, suggesting rapid renal excretion and early-onset after injection. A similar study that used ^68^Ga-DOTAVAP-PEG-P2 showed rapid renal excretion from 5–120 min [32]. Besides liver uptake observed in this study, there was no significant uptake in other organs such as lung or heart. Higher liver accumulation was detected in a previous study that used ^68^Ga-DOTA-nitroimidazole where the authors reasoned that the tracer accumulation in the liver might be due to the compound's high lipophilicity [33].

Despite the high specific activity and acceptable binding affinity of ^68^Ga-DOTA-TBIA101, imaging of* E. coli* was considered suboptimal. For this reason further matters should be addressed that might be involved in causing lower uptake in an active infection site. Whilst the advanced state of the infection duration could have likely led to a strong eradication of the bacilli in immunocompetent animals, it cannot be ascertained that this was the case with the particular amount of* E. coli* injected originally in the reported experiment. In some cases the bacterial burden following an intramuscular or subcutaneous inoculum of laboratory bacterial is almost negligible; the recovery rate from the infection site returned very low. In this way a reduced bacterial multiplication could occur and include a prolonged lag (bacteriopause) phase. More virulent strains, however, can cause massive wounds even after a short incubation period. As some depsipeptides form the active part of streptogramins, an antibiotic class of compound targeting the bacterial ribosomal activity, it could be concluded that a quiescent (homoeostatic) bacterial state is unlikely to be targeted by depsipeptide-based tracers [34]. Therefore, particularly in unestablished animal infection models, a 24-hour-old microbiological tissue culture including CFU counting is encouraged. Furthermore, bacteria are naturally equipped with surface-bound and/or secretory proteases, a considerable defence mechanism that can inactivate antimicrobial compounds like DOTA-TBIA101 and that may result in a reduced tracer accumulation, even though viable bacteria are present. For example, the outer-membrane (OmpT) protease (of an enterohemorrhagic* E. coli* strain) disunites and inactivates an *α*-helical AMP (LL-37) but cannot cleave a disulfide-bond-stabilized AMP (HNP-1) [35, 36]. In addition there are further protective shielding strategies that bacteria employ to resist antimicrobial action; two of these strategies are modulation of the host innate immune response [37] and bacterial DNA mediated downregulation of bactericidal peptides in enteric infections [38]. Furthermore, bacteria can make use of cationic capsule polysaccharides or membrane phosphate charge masking mechanism or activation of ATP-binding cassette (ABC) transporters [39].

The results warrant further preclinical imaging studies as the DOTA-conjugation to TBIA101 did not appear to compromise the TBIA101 capacity as the targeting vector. These studies should include sterile inflammation-control experiments as well as ensure viable bacteria in the target site over a shorter time span after inoculation with aerobic bacterial strains; thus, the lack of an experiment of greater depth might be the reason for the lower-than-expected target-to-nontarget ratio.

## 5. Conclusion

We reported on the depsipeptides-deriving compound ^68^Ga-DOTA-TBIA101 and its “proof-of-concept” approach to target infected muscle tissue (although in low-target-to-nontarget ratios), providing noninvasive imaging of infection using PET/CT at a late stage. As prerequisites, ^68^Ga-DOTA-TBIA101 radiolabeling, bacterial binding, cytotoxicity, integrity, and stability met the criteria to warrant the envisaged imaging studies to approve its target-to-nontarget ratio before further animal studies could commence.

## Figures and Tables

**Table 1:** ^
68^Ga-DOTA-TBIA101 radiolabeling using a SnO_2_-based ^68^Ge-^68^Ga generator.

Peptide conjugate	^ 68^Ga-DOTA-TBIA101
Number of radiosyntheses (*n*)^$^	23
Generator elution	
Total ^68^Ga-activity eluted (MBq)	1437 ± 489
Waste fraction (%)	7.1 ± 2.2
Generator use (days), (range [*n*])	198 ± 113, (11–279, [13])
^68^Ga-activity added (MBq)	1201 ± 475
Buffer solution/peptide concentration (*µ*M)	2.5 M Sodium acetate/40
Optimal pH value	3.5 ± 0.4
Temperature (°C)/duration (min)	95/10
SPE C18-unit type	
Small sample volume <0.5 mL	C18 Sep-pak light 100 mg
Large sample volume >0.5 mL	C18 Sep-pak 500 mg
SPE C18-unit elution mixture	
Standard mix (v/v)	EtOH/Saline (1 : 1)
Alternative mix (v/v)	CH_3_CN/PBS (1 : 4)
Unit desorption ratio (%)	77 ± 21 (*n* = 6)
Specific activity (GBq/*µ*mol)	12.4 ± 6
Time EOL to purified product (min)	39 ± 6 (*n* = 8)
Recovery of radioactivity (%)	102 ± 2 (*n* = 8)
Radiochemical purity	
Crude/final product HPLC (%)	>92/99 (*n* = 2)
Crude/final product ITLC (%)	>90/99 (*n* = 4)
Loss to apparatus and colloids (%)	18 ± 8 (*n* = 8)
Reproducibility	
Average %LE ITLC (range min–max)	64 ± 18.5 (16–85)
LE <25%: *n* (%)	1, (4.3)
LE 25–60%: *n*, (%)	6, (26.0)
LE >70%: *n*, (%)	12, (52.0)
End product activity half scale (MBq)^*^	668 ± 385

^$^Unless stated otherwise results are presented as mean ± SD of 23 of radiosyntheses. %LE = percentage labeling efficiency. ^*^Details are given in Section 2.

**Table 2:** Organ/tissue concentration and biodistribution of ^68^Ga-DOTA-TBIA101 in *Escherichia coli* infected mice.

Organ/tissue	Activity concentration (SUV)		Compound distribution (%ID)	
	Infected	Healthy control^*^	Infected	Healthy control
Heart	**16 ± 2.2**	0.43	9.5	8.9
Lung	**0.73 ± 0.03**	0.48	0.50	0.50
Liver	**25 ± 3.9**	14	14	14
Kidneys	**59 ± 6.3**	35	36	36
Brain	**0.90 ± 0.1**	0.55	0.55	0.57
Intestine	**0.90 ± 0.1**	0.54	0.58	0.57
Urinary tract/bladder	**34 ± 2.9**	20	21	21
Forearm muscle	**1.1 ± 0.2**	0.58	0.63	0.60
Hind muscle (CL)	**1.5 ± 0.3**	0.73	0.77	0.76
Hind muscle (*E.coli*)	**1.7 ± 0.3**	0.73	0.94	0.76
Hind muscle (CL) to forearm muscle	**1.4 ± 0.1**	NA	NA	NA
Hind muscle (*E.coli*) to background ratio	**2.3 ± 0.5**	NA	NA	NA
Hind muscle (*E.coli*) to forearm muscle	**1.6 ± 0.1**	NA	NA	NA
Hind muscle (*E.coli*) hind muscle (CL)	**1.2 ± 0.1**	NA	NA	NA

Values expressed as mean ± SEM. *E.coli* = *Escherichia coli* 22952 (*n* = 3), healthy control (*n* = 1), and CL = contralateral. ^*^The SUV values were calculated from the same region of interest area using one healthy animal.
